# Framework for processing operando neutron radiography of energy devices

**DOI:** 10.1038/s41598-025-09425-w

**Published:** 2025-07-16

**Authors:** Jongmin Lee, Eric Ricardo Carreon Ruiz, Anders Kaestner, Pavel Trtik, Markus Strobl, Pierre Boillat

**Affiliations:** 1 PSI Center for Neutron and Muon Sciences, Paul Scherrer Institute, Villigen PSI, 5232 Switzerland; 2 PSI Center for Energy and Environmental Sciences, Paul Scherrer Institute, Villigen PSI, 5232 Switzerland

**Keywords:** Energy storage, Fuel cells

## Abstract

Neutron radiography is a powerful diagnostic technique for operando studies of electrochemical devices, such as fuel cells, batteries, and electrolyzers. However, processing time-series neutron images is challenging due to high spatial/temporal resolution requirements, limited neutron flux, complex sample geometry, and low signal-to-noise ratios. Existing image processing platforms are not adequate to mitigate these issues, causing bottlenecks in data analysis and interpretation. In this work, we present our Python-based framework: neutron radiography of electrochemical devices (NeuRED). This framework integrates a robust set of image processing functions within a transparent, reproducible, and user-friendly workflow. The advantages and unique features of the framework are outlined, and demonstrations are provided for proton exchange membrane fuel cells, Li-ion batteries, and gas-liquid systems. NeuRED is a unique open-access software tool for the electrochemistry community that will contribute to the advancements of operando imaging applications in energy research.

**Supplementary Information:**

The online version contains supplementary material available at 10.1038/s41598-025-09425-w.

## Introduction

Electrochemical devices are green energy conversion and storage devices that are essential in achieving carbon neutrality. These devices consist of multiple functional layers: a pair of electrodes, an ion-conductive membrane or separator with an electrolyte, and supporting porous materials. Understanding mass and heat transport within them during operation provides key insights into material design and operational strategy that leads to improved performance and durability. Diagnostics based on neutron and synchrotron facilities are the primary methods that allow operando investigation of electrochemical devices at sufficient spatial and temporal resolutions.

Neutrons possess unique properties compared to other probes, such as photons and X-rays. Neutrons interact with the atomic nucleus, providing high sensitivity to some low atomic number (Z) elements, whereas X-rays are more effective for detecting high-Z elements. This makes neutron techniques particularly advantageous for studying electrochemical devices, where hydrogen and lithium-based reactants, products, and materials are prevalent. Neutron-based methods, such as small-angle scattering, diffraction, reflectometry, and spectroscopy, have been widely applied to study energy materials^1,2^; however, these techniques, which focus on reciprocal space analysis at usually statistical submicron scales, do not provide spatial resolution on the macroscopic scale. In contrast, neutron imaging offers spatially resolved information, unaffected by cell housing and support structures such as fuel cell endplates or battery casings^3^. Therefore, neutron imaging has been extensively used over the past 20 years to study reactant and product distributions, identifying underlying transport mechanisms in electrochemical devices^4^. The studies include water management in polymer electrolyte fuel cells (PEFC)^5–10^filling state and bubble formation in electrolyzers^11–13^lithiation and delithiation in Li-ion batteries^14–17^as well as flow and air batteries^18,19^.

In conventional neutron imaging, neutron beam attenuated by sample arrives at a scintillator, where it is converted to visible light. After being reflected by a 45º mirror and focused through an optical lens, the light is captured by a digital detector, such as a charge-coupled device (CCD) camera. In time-series imaging of an operating electrochemical device, changes in pixel values, relative to images taken at a reference state (e.g., open circuit voltage), can be quantitatively linked to physical changes using the Beer-Lambert law^20^:

1 $$I = ~I_{0} \cdot e^{{{-}\Sigma \cdot \delta }} ,$$

where $$\:I$$ is the intensity of the transmitted neutron beam, $$\:{I}_{0}$$ is the intensity of the incident beam, Σ [cm^− 1^] represents the attenuation coefficient, and δ [cm] is the sample thickness. In the final processed images, pixel values represent transmission ($$\:\raisebox{1ex}{$I$}\!\left/\:\!\raisebox{-1ex}{${I}_{0}$}\right.$$), optical density ($$\:\varSigma\:\cdot\:\delta\:$$), or thickness (δ) depending on the available information and purposes of analysis.

A general challenge in neutron imaging is attributed to limited neutron flux, resulting in longer exposure time (typically orders of 10s) to collect signals with sufficient signal-to-noise ratio (or counts) to allow quantitative analysis^21^. In typical materials and objects studied using neutron imaging, such as concrete, metal alloys, fossils and cultural heritage, the regions-of-interest are on the mm-to-cm scale, and the timescale of physical and chemical phenomena is much greater than the image acquisition time (e.g., metal corrosion). In contrast, electrochemical device investigations require the highest spatial and temporal resolution to study multiple processes occurring in thin layers (orders of tens to hundreds of micrometers and tens of seconds). Moreover, housing material, gas/liquid inlet and outlet, temperature control, and electrochemical control lead to further limitations including blurring due to reduced L/D (where L and D are defined as the aperture-to-sample distance and aperture diameter, respectively)^22 ^and increased contributions of biases (scattering) and outliers (gamma spots) caused by the neutron matter interaction with samples and infrastructure interfering with the quantitative analysis. The outlined issues are inherent to neutron imaging beamlines across various facilities, regardless of source type (spallation, high-flux reactor, or laboratory source). While no standard configuration exists, combinations of camera, scintillator, lens, and detector setup generally result in comparable image quality for a given neutron flux. This underlines the universal importance of systematically identifying and addressing imaging artefacts to ensure data integrity and maximize research output.

Despite significant efforts to advance neutron imaging of electrochemical devices, there is currently no dedicated software developed for this purpose. Open-source platforms such as Mantid^23, ^KipTool^24, ^and ImageJ^25^ provide user-friendly environments for general image processing and visualization. However, they are not optimized for handling time-series neutron images of electrochemical devices for the reasons below.

- The processing functions in these platforms do not fully account for the specific characteristics of neutron images of electrochemical systems. These images often capture physical phenomena occurring within stacks of highly contrasting layers, typically only a few hundred micrometers thick, resulting in limited number of pixels. Filtering, registration, and alignment functions designed for biological or other applications are often not suitable.
- The application of image processing functions requires systematic parameter testing. In existing software, this step is not always intuitive or transparent, resulting in high levels of inconsistency.
- Generally, there is no built-in documentation of the processing pipeline within image data, which hinders the reproducibility and tracking of image handling. The extensive maneuver required increases the likelihood of human error and inaccuracies in the results.
- Time-series radiographs of electrochemical devices involving comparisons between reference-state and operando images to isolate changes in the system. Reference images need to be fully processed and inspected prior to comparing to operando images. A dedicated program designed around this data structure would streamline image processing more efficiently.

In this work, we present the Neutron Radiography of Electrochemical Devices (NeuRED) framework, developed in Python, originally designed for processing neutron image series of operando fuel cell studies^26–28^. This framework has been successfully extended to other time-series neutron radiography applications, including Li-ion batteries and pressurized gas-liquid systems. Moreover, NeuRED framework is compatible with operando X-ray radiographs, as well as pre-processing of tomographic projections. Rather than serving as an introductory manual for the software, the aim of this work is to compile scientific approaches to neutron image processing and demonstrate their capabilities through practical examples.

## Results and discussion

This section outlines the key features of the NeuRED Framework and demonstrates its application to various types of time-series neutron imaging of electrochemical devices. The first example focuses on fuel cells, for which the framework was originally developed to quantify water content in both through-plane and in-plane imaging configurations. The second example is a CR2032 coin cell battery (20 mm diameter, 3.2 mm height) that is widely used in both research and commercial applications. The final example is a pair of cylindrical cells containing deuterated organic liquids, such as methanol, ethanol, or p-xylene, along with hydrocarbon gases under varying temperatures and pressures.

### NeuRED framework and its advantages

#### Functions and pipeline for image processing

The NeuRED Framework is designed to provide a versatile and transparent way to manage and create image processing pipelines. Users can choose image processing steps from *proc functions* and group them in order, defined as a *sequence* that serves as an input to processors (*single_proc*, *batch_proc*,* single_merge*). On the other hand, background functions, which are not directly involved in image manipulation (e.g., read, write, and display), are nested in the *modules* as Python files.

**Fig. 1:**
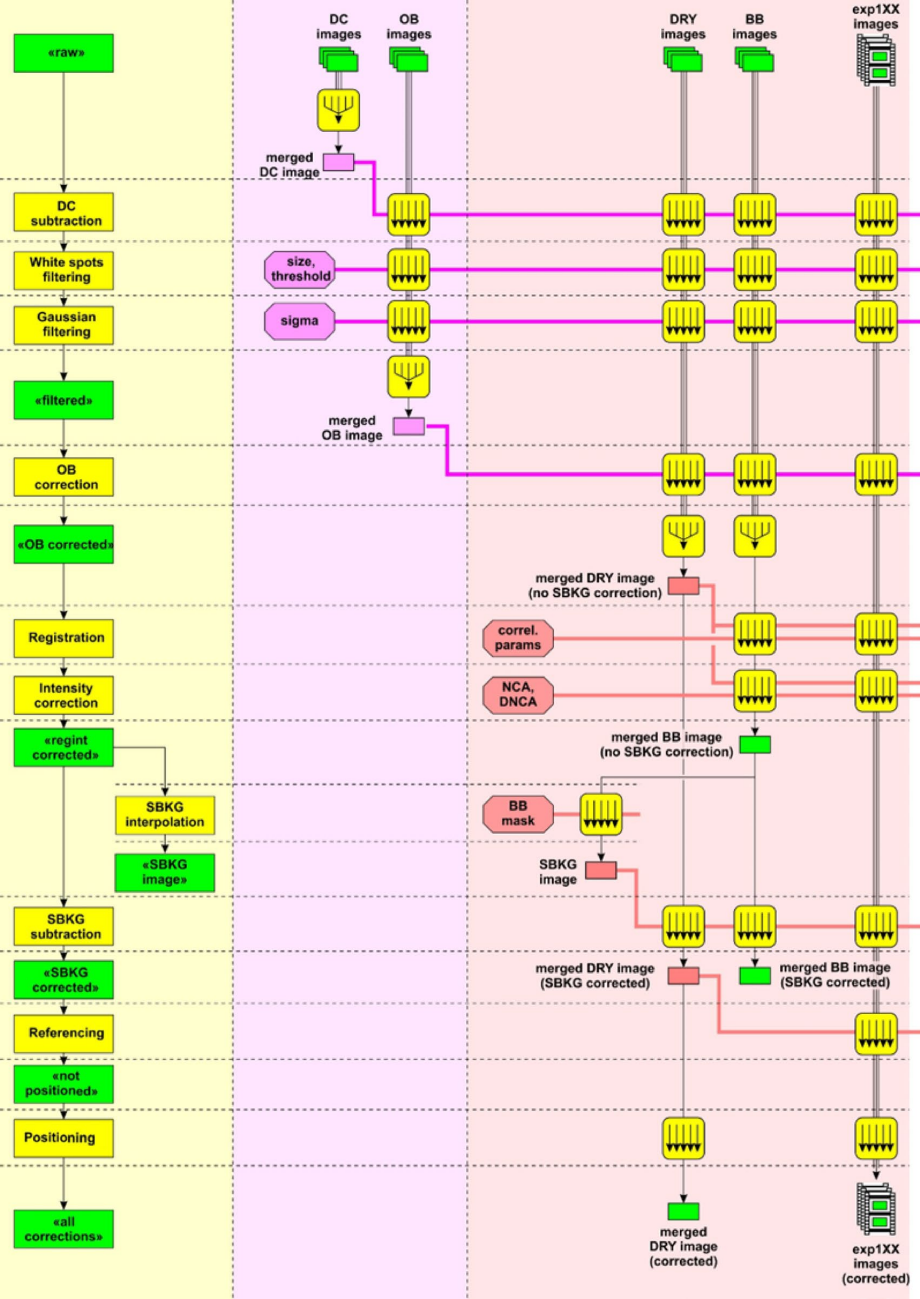
Image processing pipeline for operando fuel cells. The acronyms used are: reference (ref), dark current (DC), merge (mrg), white spot (WS), black body (BB), non-changing area (NCA), dark non-changing area (DNCA), and registration region-of-interest (reg_roi). On the left most column (light yellow background), the yellow boxes represent functions, whereas the green boxes indicate the stage of workflow.

The processing steps for two-dimensional (2D) time-series neutron images are well known^23–25^. In the NeuRED Framework, we optimized the pipeline and implemented new features specific to electrochemical devices. These include three-dimensional (3D) white spot filtering instead of 2D filtering per image, multiple region-of-interest (ROI)-based image registration (translation, rotation, and skew), and intensity correction. The underlying principles and some demonstrations of these basic methods have already been discussed in the thesis of Boillat^20^. The main image processing steps are summarized below and in Fig. 1.

- Dark current (DC) correction: The read-out noise is subtracted from all images.
- White spot (WS) filtering: Gamma rays contaminating the detector result in clusters of abnormal pixels with high (often fully saturated) counts, referred to as white spots. These spots are identified by a threshold, investigating 99% confidence interval (CI) on the right tail of the fitting. The WS values are substituted by 3D median filtering involving surrounding pixels and adjacent images, where the size of the filter is an input parameter. This step is particularly important for images acquired at low-flux facilities, such as laboratory neutron sources, where gamma radiation has a pronounced impact on image quality.
- Gaussian filtering: Statistical noise is suppressed by applying a Gaussian filter. However, this step introduces geometric blurring; therefore, the function parameter (sigma) should be optimized to balance statistical noise reduction with the retention of feature sharpness.
- Open beam (OB) normalization: OB images, also known as flat fields, are acquired without sample. By dividing sample images by open beam images, transmission images ($\:I/{I}_{0}$) are produced where non-uniformities in the beam profile and local detector efficiency are eliminated.
- Registration: In case a sample moves during a time-series measurement, a set of ROIs are chosen to track the movement of specific features. An inverse matrix is computed to perform affine transformations (translation and shearing).
- Intensity correction: Neutron flux at the sample position may vary depending on the operation of the source and its stability. To compensate for incident beam intensity differences among images, intensity (dose) correction is executed by matching average values of a set of non-changing areas (NCAs, gain) or dark non-changing areas (DNCAs, offset) to the reference image.
- Scattered background correction^33^: Neutron images are significantly affected by several modes of scattering: (1) light scattering in the detector, (2) neutron scattering within the detector, and (3) neutron scattering due to the sample (reciprocal information typically captured via using small- and wide-angle neutron scattering techniques). To account for scattering background, a matrix of black body (BB) dots, composed of highly neutron absorbing materials, is placed in front of the sample to produce BB images. Since the pixels corresponding to the dots should have zero transmission values (or values corresponding to those of the dark currents), the scattered background can be estimated by interpolating the values behind the dots throughout the field-of-view (FoV). The lower the transmission values, the more strongly images are affected by scattered background, as is common in low-flux laboratory neutron imaging.
- Referencing: Operando images are divided by the reference images to evaluate the changes in the system according to the Beer-Lambert law. The reference images are typically obtained at the beginning of the experiment. For example, for fuel cells, the reference images are acquired with partially or fully humidified nitrogen gas (referred to as dry).
- Thickness conversion: The transmission values in the referenced images are converted to the thickness of the known species for the given attenuation coefficient.
- Positioning: For image analysis, it is beneficial to align macro features of the sample (e.g., membrane electrode assembly, channels, and gas diffusion layer in the fuel cells) with the horizontal or vertical axes. In this step, an image is rotated by a user input angle. In case images were obtained using a tilted scintillator setup (anisotropic magnification), affine transformation can be applied to correct for shear distortion^20^attributed to optics misalignments.

#### Global variable approach: param and processors

In the NeuRED framework, variables are defined globally as *params*, regardless of their data type (e.g., strings, integers, floats, lists, dictionaries, or images). Parameters can also be grouped under a scope, for example, using *set_general_scope (“cell1”)* where “cell1” can be replaced with “cell2” and so on for batch processing. The *Modules* contain various functions for importing, exporting, and managing background processes for different types of data and designated purposes.

An engine that performs the processing for the chosen functions and data is called a processor. The framework provides four processors: *test_proc*,* simple_merge*,* single_proc*,* and batch_proc*. For each processor, input and output must be defined in the form of *param*, and functions need to be grouped into *Seq* in the order of application. For more details, readers may refer to Supplementary 2 where example codes are provided with detailed explanation.

#### Parameter optimization and test display

**Fig. 2:**
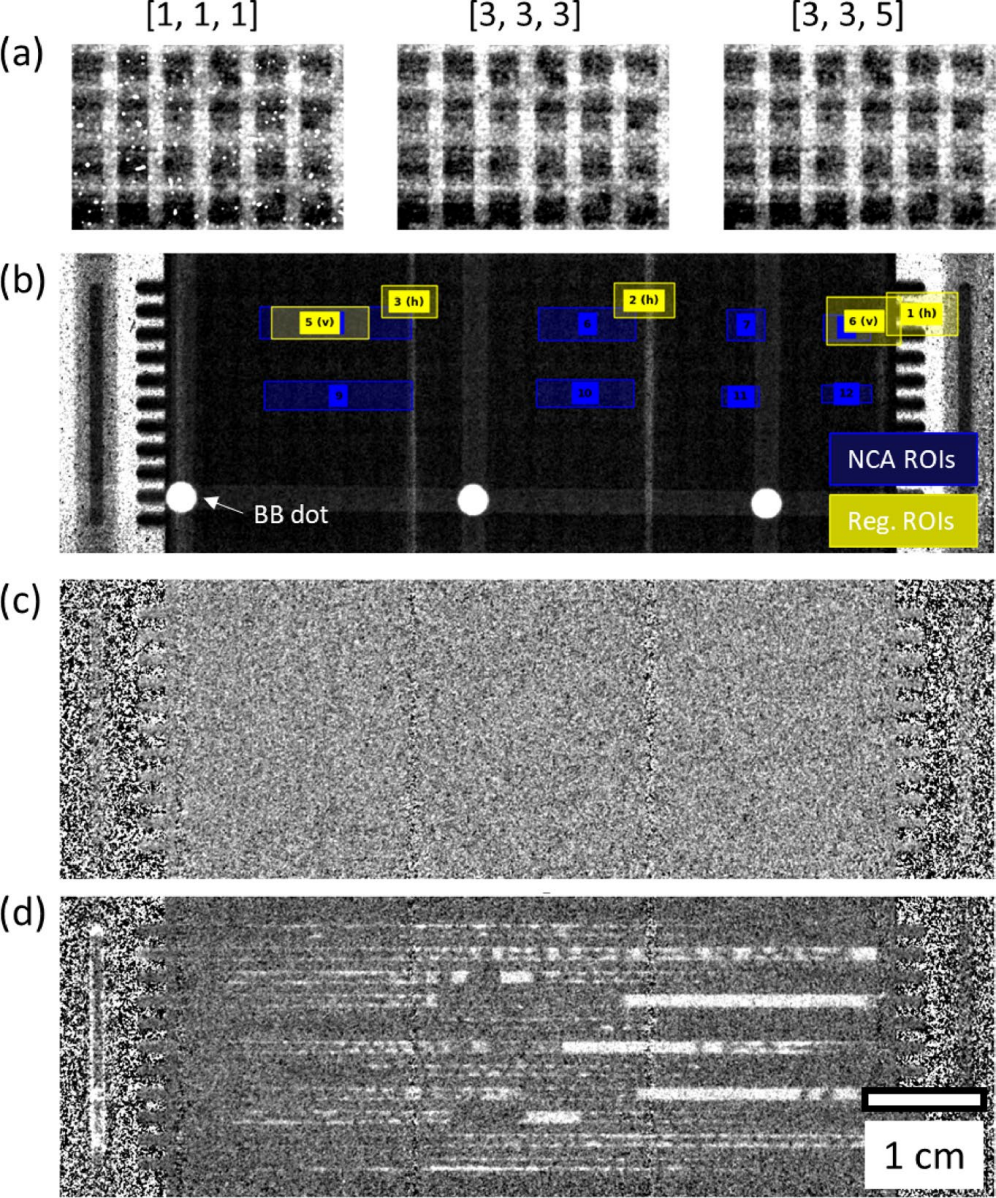
Through-plane images of fuel cells at various image processing steps. (**a**) Raw intensity image with varying white spot filter sizes: from left to right, ws_filter_size = [1, 1, 1], [3, 3, 3], and [5, 5, 5]. (**b**) Transmission image annotating NCAs (blue) and registration ROIs (yellow) used for image alignment, with horizontal (h) and vertical (v) orientations specified. Fully processed water thickness images of operando fuel cells under (**c**) dry and (**d**) wet conditions, where white regions indicate areas of water accumulation.

The input parameters, such as *ws_filter_size* (3D filter) and *sigma* (Gaussian filter), need to be optimized for each sample and setup. For example, increasing *ws_filter_size* improves statistical accuracy, but it significantly extends processing time and reduces the number of usable images at the start and end of the dataset. Parameter testing is done through *test_proc*, where the selected parameter *(test_param)* can be evaluated for various values (*test_values*). An example of testing *ws_filter_size* with different values is shown in Fig. 2 (a).

Additionally, *test_proc* can be used to display results during the process. Figure 2 (b) shows the results of through-plane fuel cell images. When *test_proc* is applied to image stacks as input, it processes the images by parsing through the list in the source folder, selecting images from the middle of the stack, and applying the chosen processing steps before displaying the results. Furthermore, various regions of interest (ROIs), such as cropping, registration, and intensity correction, can be visually displayed for confirmation as shown in Fig. 2 (c). The fully processed image (water thickness map in this case) can be also displayed, Fig. 2 (d).

#### Process tracking: metadata headers

A key advantage of the Flexible Image Transport System (FITS) over the Tagged Image File Format (TIFF) is incorporation of metadata where information can be written in headers. The NeuRED framework is compatible with other image format inputs, but output images are in FITS files.

**Fig. 3:**
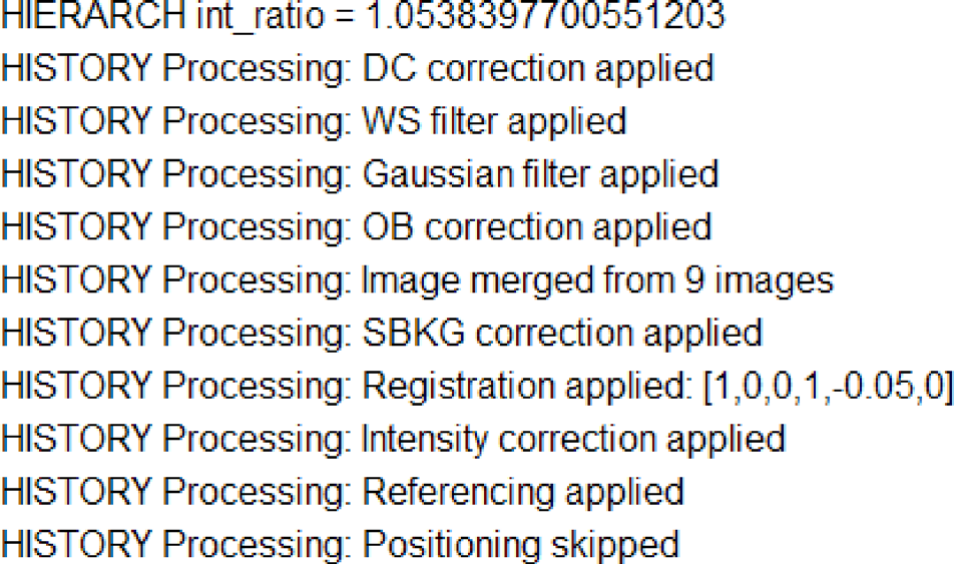
Example headers of processed images in FITS format, documenting the applied processes and relevant parameters. The HIERARCH int_ratio entry indicates the multiplication factor applied to the image relative to the reference image. In the registration line, the list represents a conversion of an identity matrix that shows displacement in the horizontal and vertical directions: [1,0,−0.05;0,1,0] indicating a subpixel shift in the horizontal alignment.

The *Modules* in the NeuRED framework are designed to interact with these headers; processes and parameters (when debugging is enabled) are recorded at each step in the order of execution. A sample header is shown in Figure 3 . If an image or parameter is missing for a specific processing step (e.g., angle input for positioning step), the framework annotates it as “skipped.”

#### Interactive user input: magic selector

One of the time-consuming and error-prone tasks in image processing is the manual input into the program. The NeuRED framework, while designed to minimize the need for repetitive manual inputs, is not entirely free from such tasks. We have developed the *magic selector* (*msel*) to facilitate the import and export of images, directories, and regions of interest (ROIs). The *magic selector* is embedded directly within a Jupyter Notebook cell and allows the tracking of input parameters progressively.

**Fig. 4:**
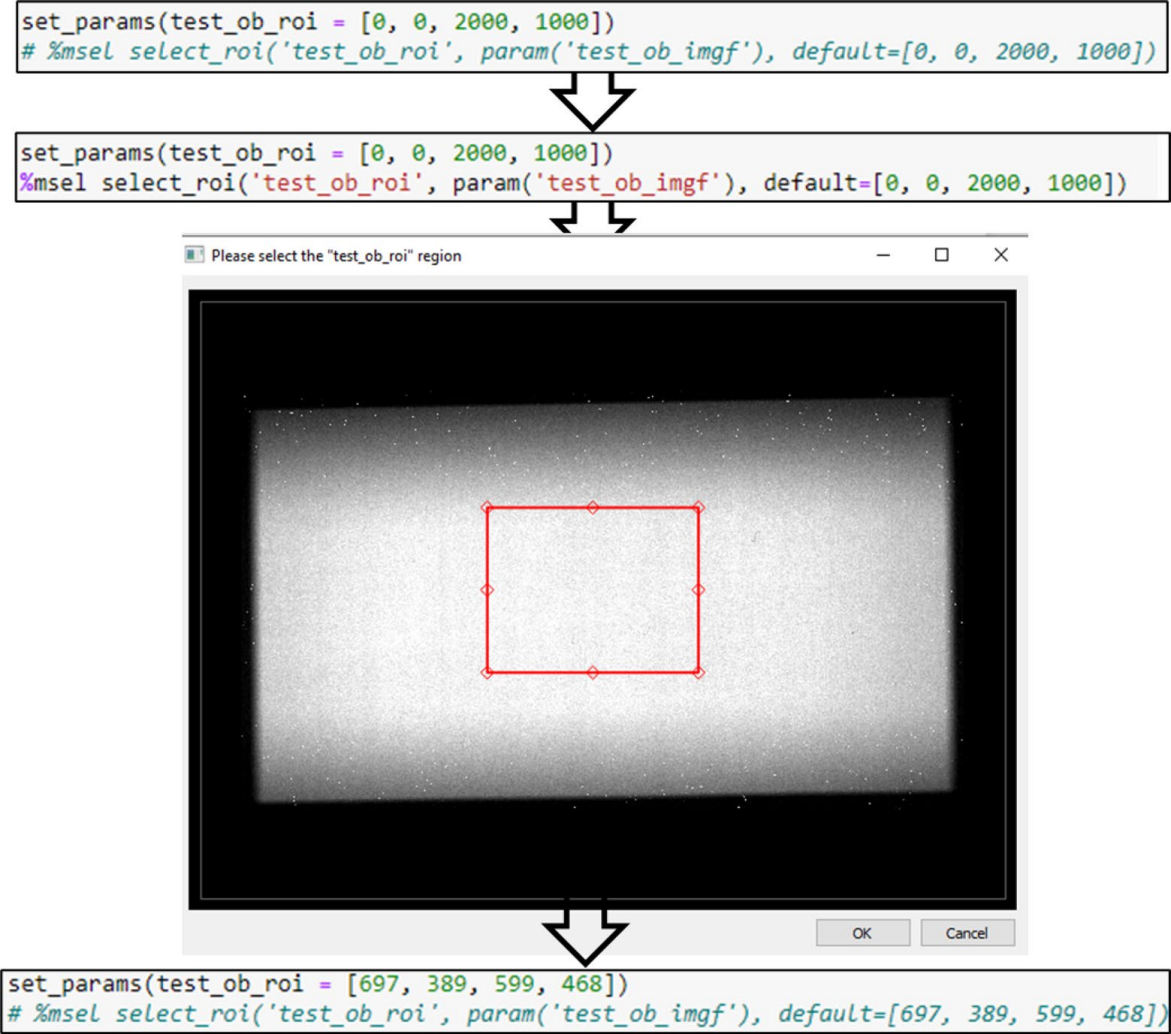
Magic selector for choosing the region of interest (ROI). The magic selector is initiated by running the cell after uncommenting the second line containing %msel. A pop-up window displaying the image (param(‘test_ob_imgf’)) appears with the default ROI.

Figure 4 illustrates the use of *msel* to select the ROI for the parameter *param(‘test_ob_roi’)* from the image *param(‘test_ob_imgf’)*. The typical structure of an *msel* unit consists of two lines: the first line sets the parameter, and the second line is initially commented out with a #. Running the cell after uncommenting the second line triggers the magic selector, which opens a separate window displaying an image (or a file explorer for directory or file selection). After selecting, the value in the first line is updated accordingly. Re-running the cell secures the entry of the value into the designated parameter. The magic selector is compatible with various input types essential for image processing, including *select_file*,* open_fits*,* save_fits*,* select_directory*,* select_roi*,* and select_multiple_rois*.

### Demonstration of NeuRED framework

#### Polymer electrolyte fuel cells

Through-plane neutron imaging is the most common method for investigating PEFCs in a single or short-stack cell configuration, with an active area size up to several hundreds of cm^2^. Since neutrons traverse through all components including bipolar plate, gas diffusion layer (GDL), and membrane electrode assembly (MEA), it is ideally used in studying the overall water distribution within flow channels.

**Fig. 5:**
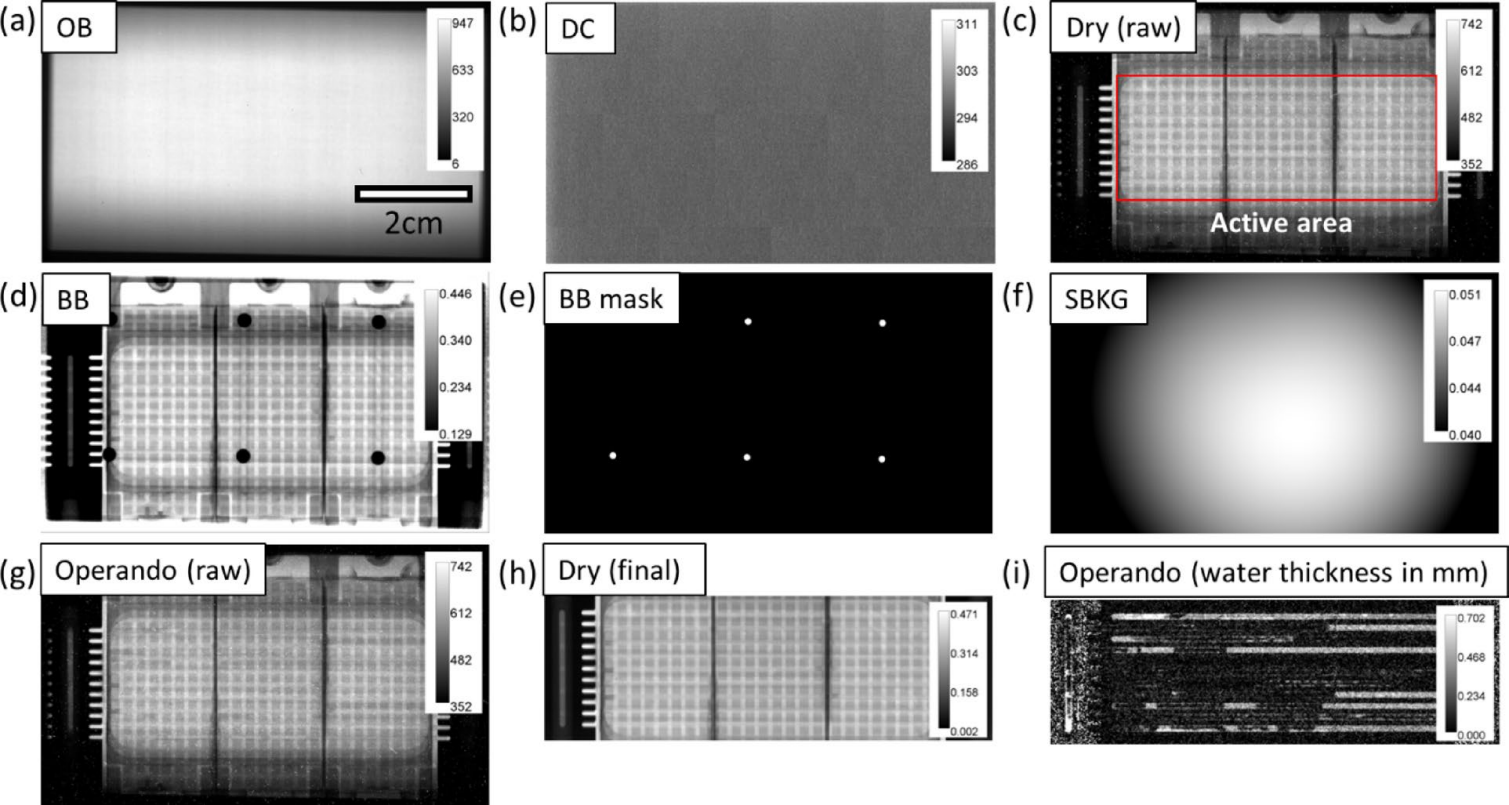
Images of a fuel cell in the through-plane direction. The NeuRED Framework: (**a**) merged open beam image after filtering, (**b**) merged dark current image, (**c**) individual raw image at the OCV state, (**d**) merged black body image, (**e**) mask image for black body dots, (**f**) estimated scattered background based on BB and BB mask images, (**g**) individual raw image during operation, (**h**) final dry image (all processes implemented except referencing and thickness conversion), and (**i**) operando water thickness image. In (**a**–**c**,** g**), pixel values are in digital counts. In (**d**), (**f**), and (**h**), transmission values are provided with respect to the OB image. Water thickness values are present in (**i**).

Figure 5 displays through-plane images of a PEFC at various stages of the NeuRED Framework implementation. Heat flux sensors and thermoelectric coolers in the custom hardware introduce grid-like patterns superimposed over the active area. Nonetheless, the transmission through the active area ranged from 0.3 to 0.4 as seen in the Dry image, enabling water quantification below the mm-scale. The scattered background values (SBKG) range from 0.04 to 0.05, corresponding to 10–15% of values in the same area of the BB image. As discussed in our previous works^21,29 ^SBKG corrections enhance sensitivity for detecting small quantities of water. The final dry and operando images, after cropping, are presented in Fig. 5 (h) and (i). In the final operando images, the quantity of water as well as shape and location in the channel can be analysed. Raw images of the fuel cell in Fig. 5 are provided as demonstration or training dataset, which can be downloaded in Zenodo (see Methods: Data Availability).

**Fig. 6:**
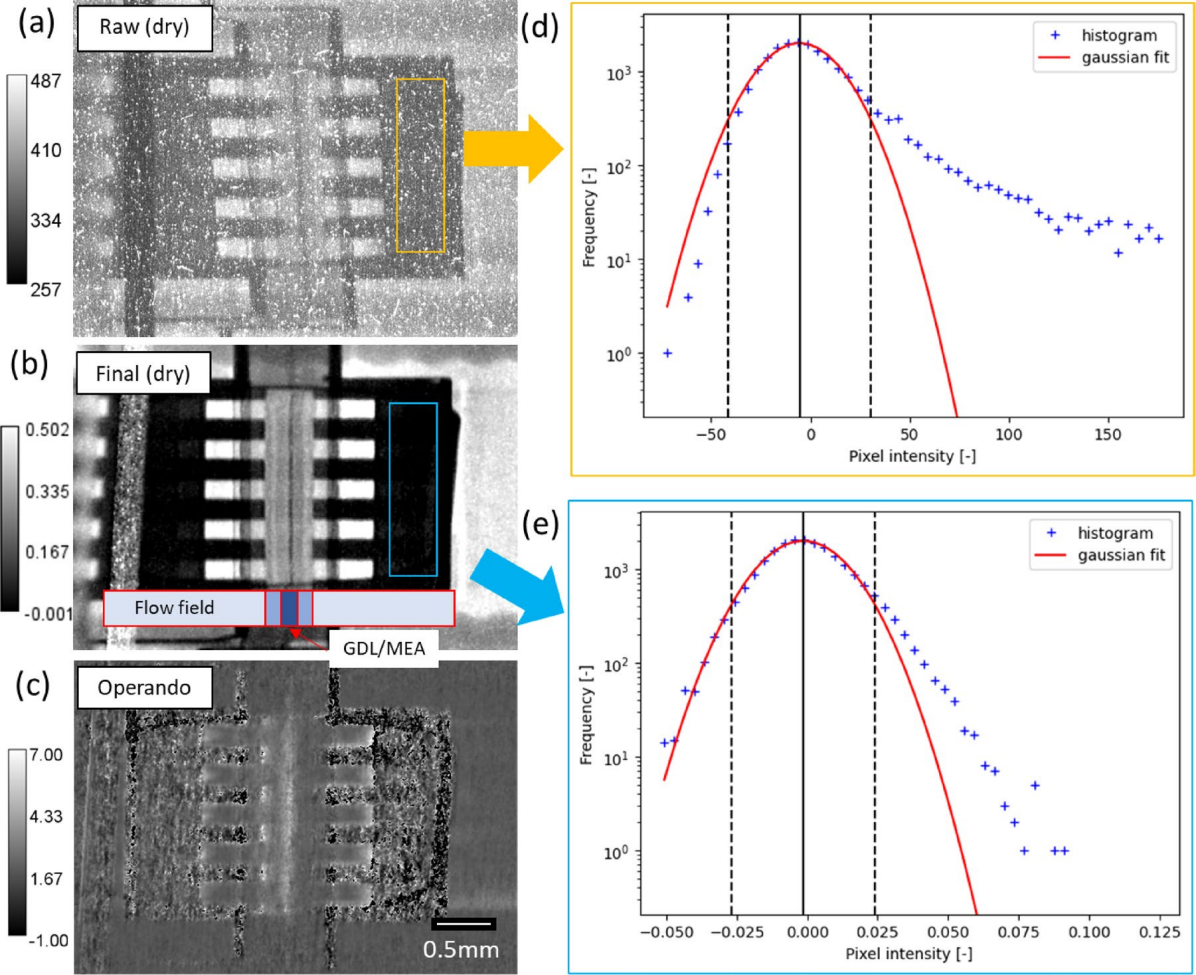
In-plane neutron images of the fuel cell with anisotropic magnification. (**a**) Single raw image under humidified N_2_ gas (referred to as the dry image); (**b**) Final processed dry image (after SBKG correction), averaged over 59 images; (**c**) Processed operando image, averaged over 3 images; (**d**,**e**) Histograms of the selected regions in (**a**) and (**b**), demonstrating the effectiveness of white spot removal. The dotted lines in (**d**) and (**e**) indicate the 99% confidence intervals. The calibration bars in (**a**–**c**) represent counts [-], transmission [-], and water thickness [mm], respectively. Pixel intensities in (**d**) and (**e**), therefore, represent counts and transmission, respectively. In (**c**), negative water thickness values appear in low-transmittance regions corresponding to the gold-plated stainless steel flow field plates.

As opposed to through-plane imaging, in-plane imaging allows for water quantification within individual layers. Figure 6 shows in-plane images of a cell in a multicell fixture, which is capable of operating six differential cells (approximately 1 cm² active area) simultaneously. A differential cell is referred as a one-dimensional (1D) cell where local variations in operating conditions (e.g., temperature and reactant distributions) are prevented, which is optimal for elucidating performance change with varying materials. A specialized imaging setup with a tilted scintillator was employed^20,30 ^achieving an enhanced effective spatial resolution in the 10 μm range in one direction. This setup is optimized for studying layered structures, such as those in fuel cells. Assuming two times finer resolution, the anisotropic magnification results in a 2^2^ times increase in exposure time (2 times for reduced flux over area, 2 times for geometric focus), rather than 2^4^ times for magnification in two directions (2^2^ times for reduced flux over area, 2^2^ times for geometric focus). This enables reasonable exposure times of under 30 s for the differential cells at the ICON and NEUTRA beamlines in PSI. However, the exposure time is significantly longer compared to through-plane images. This leads to an accumulation of white spots, as seen in Fig. 6 (a), which need to be filtered carefully without compromising spatial resolution and accuracy.

White spots are distinguished by a threshold determined by *ro_auto_threshold* function based on 99% confidence interval (CI) constraint. To proceed with the filtering process, two user input parameters are required: the median filter size and the sigma for the Gaussian filter. For each parameter, *test_proc* is executed to visually confirm the optimal value. The effectiveness of the filters is demonstrated in Fig. 6 (d) and (e), which compare histograms of the flow field region for the raw image and the final image. To optimize the determination of the white spot threshold, a region with relatively low transmission, such as the gold-plated stainless steel flow field, should be selected to effectively distinguish white spots from the true sample signal. Pixels with grey values above the 99% CI in the raw image are successfully suppressed in the final image after filtering and averaging. As a result, individual layers within the differential cell are visible. The dark line at the center of the MEA represents the membrane, which strongly attenuates neutrons due to hydrogen in the sulfonic acid groups and bound water. A key challenge in operando in-plane radiography of fuel cells is membrane swelling, which causes material shifts and artificial water signals during image normalization, especially at the MEA-MPL interface. This effect can be reduced by pre-conditioning the membrane with fully humidified N_2_ gas after purging residual liquid from prior experiments. As a result, so-called dry images are acquired with humidified membrane without liquid water in the system. Similar to the final operando image in Fig. 5, the in-plane water quantity can be determined, which reveals significant water accumulation under the rib due to condensation and within the membrane due to water uptake.

#### Half-cell Li-ion coin battery (CR2032)

In neutron imaging studies of Li-ion batteries^31–33, ^custom cells are often used with electrolytes containing deuterated organic solvents. However, for researchers with limited expertise in neutron science or hardware design, applying neutron imaging to their materials of interest can be challenging. To provide a more accessible example for the broader community, in-plane images of an in-house-built CR2032 cell with commercial components were selected for the processing demonstration. Unlike fuel cells that achieve a steady state between reactant (water) production and removal within 10–20 min of initiating galvanostatic operation, Li-ion batteries exhibit continuous changes in lithium distribution throughout multiple cycling events, with lithiation of the negative electrode during discharge and delithiation during charge, spanning hours or even days. Consequently, a key challenge in managing long time-series of battery data is compensating for beam fluctuations over the imaging period.

**Fig. 7:**
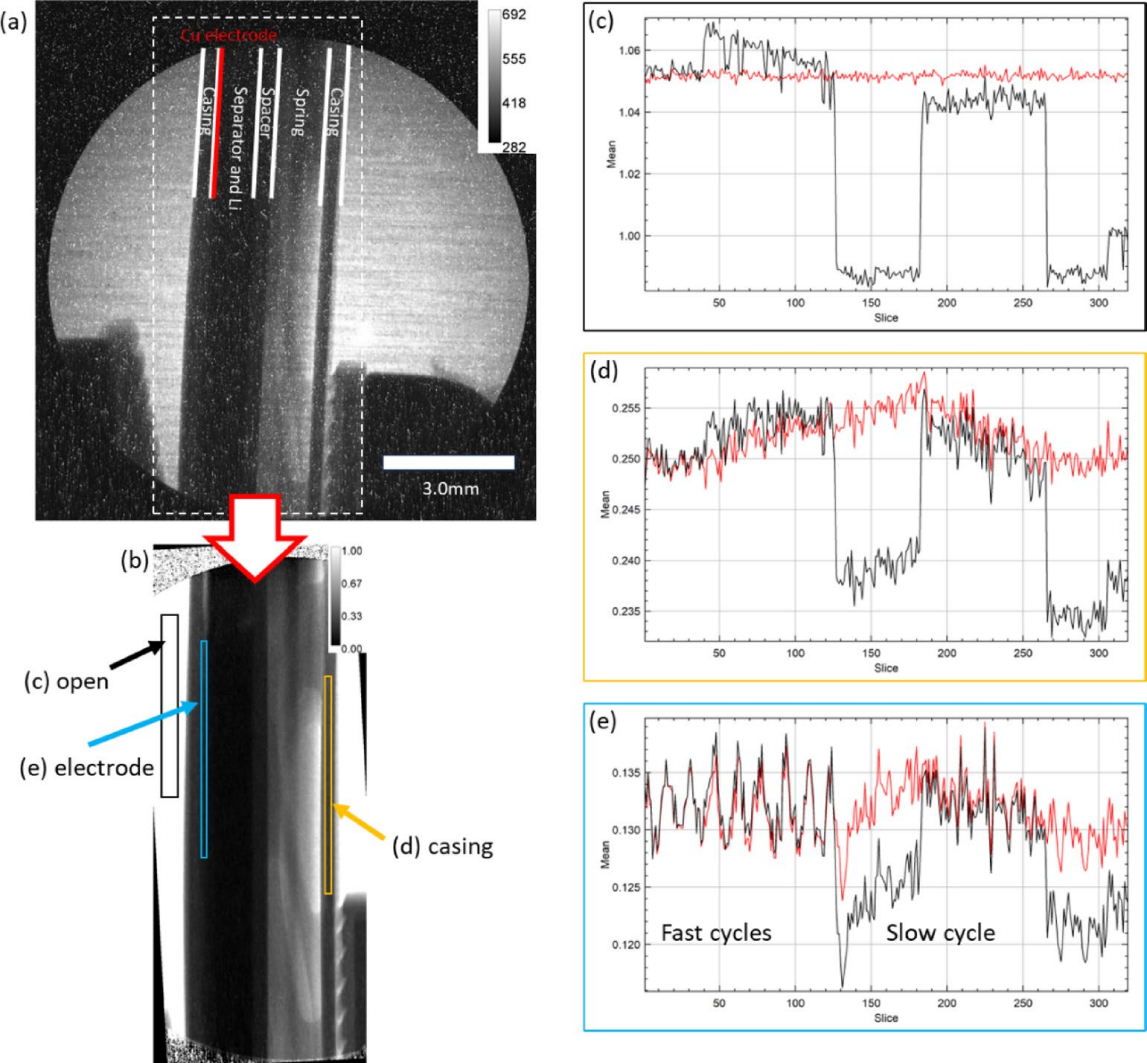
(**a**) Raw image of a CR2032 Li-ion half-cell battery acquired using a high-resolution taper setup in in-plane configuration. The red line marks the position of the Cu electrode. Since the battery (d = 20 mm) was larger than the FoV (ca. 10 mm), only the center part of the battery was imaged. The dotted white box indicates the cropped region shown in (**b**). (**b**) Average of 10 fully processed images over 5-min period (30 s exposure per image) where SBKG correction, referencing, and thickness conversion steps were skipped. Black, yellow, and blue boxes correspond to open beam, stainless steel casing, and copper electrode regions, respectively. (**c**) Average transmissions in the open beam region before (black) and after (red) intensity correction over 26 h. (**d**) Average transmissions in the casing region. (**e**) Average transmissions in the electrode region. The saw-tooth patterns in the first 130 slices represent Li plating and stripping on the copper electrode, (80 min per cycle). For the slices after 130, slow cycling was imposed.

In Fig. 7(a), a raw image of the half-cell Li-metal CR2032 coin cell is shown. The cell consisted of a Cu electrode, a fiberglass separator, a natural Li metal disc, and electrolyte (protonated ethylene carbonate and deuterated dimethyl carbonate in a 1:1 volume ratio). Conventional anode (carbon-based) and cathode materials (LiFePO_4_ or NiMnCoO_2_) are relatively neutron-transparent, and neutron imaging studies have provided valuable insights^34^. However, a key limitation remains the spatial resolution in the in-plane configuration when using thin, commercial electrodes. To enhance lithium signal for improved detection limits, enriched^6 ^Li can be used; however, it is predominantly available only in metallic form and is impractical for most electrode materials. Additionally, microporous polymer separators containing hydrogen strongly absorb and scatter neutrons and should be used with caution. Polytetrafluoroethylene (PTFE) offers excellent neutron transparency and has been explored as a separator material. However, microporous PTFE membranes generally have lower mechanical robustness and are more difficult to manufacture with controlled porosity and thickness compared to conventional polyethylene-based separators.

The high-resolution fibre optics taper setup provides a circular FoV of approximately 10 mm, which limits the visualization to the central portion of the 20 mm battery. In this setup, a bundle of fine boron glass fibres, with gradually increasing diameter, forms a truncated-cone shaped solid (fibre optic taper) placed between the scintillator and camera box, functioning as an image magnifier^35^. Key components such as the stainless-steel housing, spring, and spacer can be clearly identified, whereas the Li metal and fibreglass separator (containing boron) cannot be distinguished due to low transmission levels. The thickness of the copper electrode was 15 μm, which is at the limit of the effective spatial resolution.

The processed image in Fig. 7 (b) demonstrates effective white spot removal and background correction, applied consistently across all individual images captured over 26 h of operation. However, the overall beam intensity was observed to be unstable during operation, manifested as low transmission in slice 125–180 and 265-end, a transmission jump at slide 40, and a gradual decrease from slide 40 to 125 in Fig. 7 (c), a black line. In the open beam region, the transmission difference between high and low intensity periods exceeds 5%, while in the copper electrode region, it was approximately 1%. Although this 1% contrast may seem negligible, it significantly affects Li content analysis considering that the transmission difference between the charged and discharged states is less than 0.5%, as shown in the saw-tooth patterns observed during fast cycling in Fig. 7 (e).

To account for beam instability, the NCA in the open beam region (black box) from slice 1 was selected as the reference image to perform intensity correction for all subsequent images. The red line in Fig. 7 (c) shows the average transmission values after correction, where the large shifts in the black line are mitigated. Similarly, the average transmissions in the casing, Fig. 7 (d), and electrode region now exhibit smooth profiles throughout the operation. The selection of the NCA is critical for accurate intensity correction. Custom hardware for neutron imaging should be designed with high-transmission and uniform thickness regions, positioned near the sample active area, which can be designated as the NCA. If the sample does not contain dedicated regions as in images of a CR2032 battery, it is preferable to select an open beam area adjacent to the electrode rather than the casing or electrolyte regions. This minimizes spatial heterogeneity with in the NCA and reduces the influence of potential physical changes during cell operation such as swelling and gas bubble formation.

As an alternative to NCA-based correction, ring current (for spallation sources) or reactor power (for reactor sources) information may be used for dose correction if recorded in the image metadata. However, these values reflect only the global neutron production and do not capture local beam variations arising from collimation, shutter operation, moderator state, or port geometry. NCA-based correction therefore remains the preferred method for neutron imaging applications.

#### Gas-liquid system

**Fig. 8:**
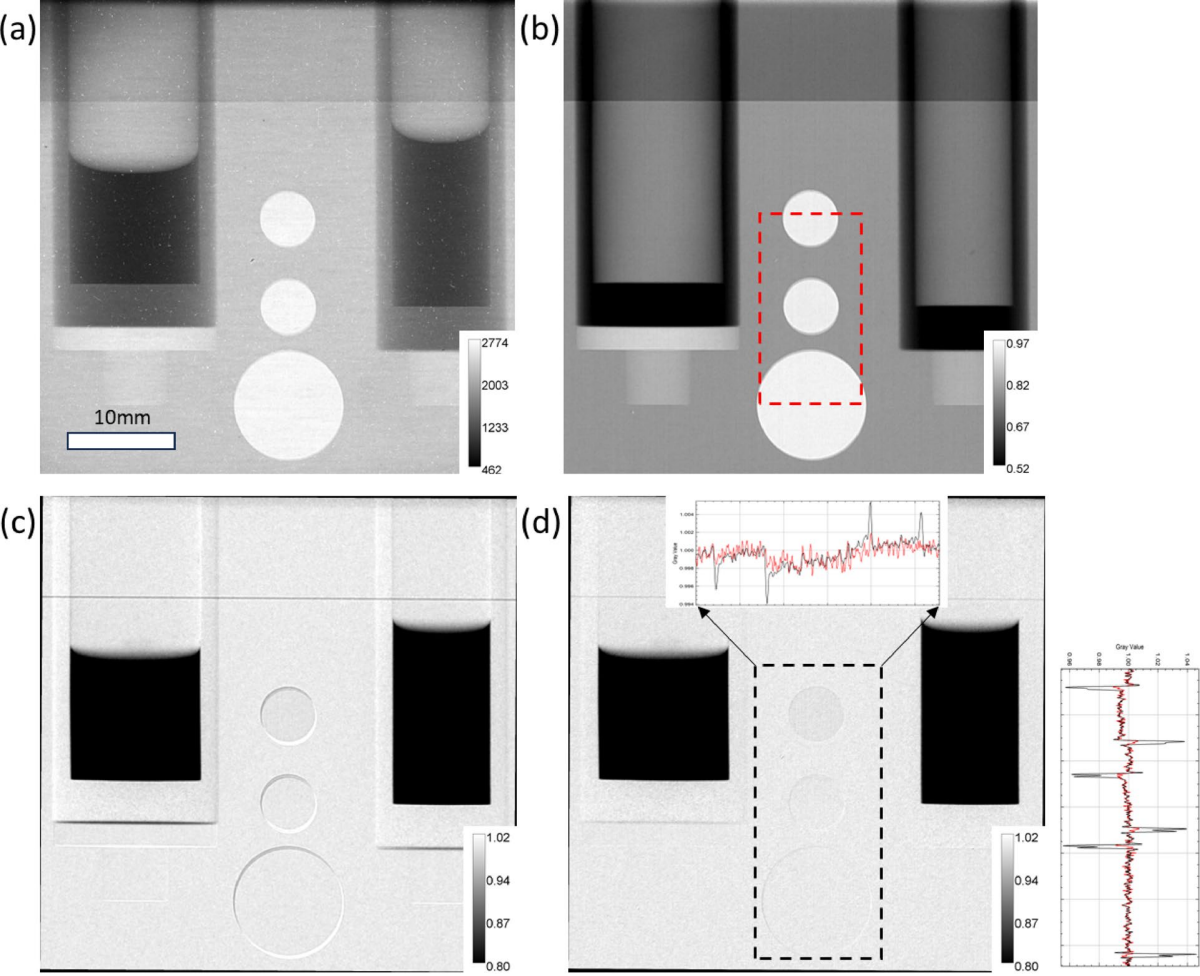
Images of two Ti cylindrical cells (left: 15 mm, right: 12 mm outer diameter) containing deuterated liquid ethanol and hydrogen gas under ambient pressure and temperature: (**a**) raw image; (**b**) average of processed empty cell images, with the dotted red rectangle indicating the ROI used for registration; (**c**) average of fully processed image without registration; and (**d**) average of fully processed images with registration. In (**d**), the dotted rectangle annotates the area where horizontal (above) and vertical (right) profiles are plotted. The black and red lines indicate before and after registration, respectively.

We have recently pioneered the application of neutron imaging to investigate the interactions between organic liquids and hydrocarbon gases (Fig. 8). Relevant industrial applications include conventional oil and gas processing, their transport and storage, as well as hydrogen storage technologies, such as liquid organic hydrogen carriers. By analyzing time-series neutron radiography under varying temperature and pressure conditions, physical and chemical properties (including surface tension, contact angle, diffusivity, solubility, and swelling) can be systematically derived. The measured values are consistent with experimental references and molecular dynamic simulations^36,37^. This one-pot neutron imaging approach enables simultaneous measurement of multiple properties; however, the accuracy and precision of values are critically dependent on the quality of image processing.

To isolate gas-liquid parts during image processing, it is preferable to normalize operando images by using empty (dry) cell images rather than open beam images. This approach minimizes the background influence of the housing material and cylindrical cells, leading to improved gas-liquid meniscus detection and enhanced contrast for hydrocarbon gas absorption in the liquid. However, a challenge associated with referencing using empty cell images originates from apparatus movement induced by the application of the gas pressure (up to 100 bars) and temperature change (from − 10 to 60 ºC). While the thermal expansion or shrinkage of metallic components is negligible for the effective spatial resolution of 50 μm, shifts in sample position can occur. Moreover, acquiring empty cell images involves extensive effort of maneuver, including disassembly, cleaning, and reassembly of the cells, followed by pitch and yaw alignment on the sample stage with respect to the neutron beam.

In Fig. 8, the effectiveness of the registration step of the NeuRED Framework is demonstrated. During the transition between operando and dry image, the apparatus was subjected to two temperature (20 and 40 ºC) and pressure (between 1 and 100 bars) cycles, resulting in horizontal and vertical misalignments that are manifested as sharp changes in transmission, particularly at material edges. By applying registration, where operando images are shifted to align with features in the ROI of the empty cell image, transmission artifacts are effectively suppressed. For effective registration, the ROI should contain pronounced features, such as distinct edges, contours, and patterns, such as through-holes shown in Fig. 8, which are specifically designed to aid in aligning the sample perpendicular to the beam and facilitating registration. This correction significantly enhances the detection of the liquid-cell interface, which is critical for accurate measurement of the contact angle. Additionally, aligning all operando images across varying conditions provides an advantage for further data processing (beyond the scope of the Framework), eliminating the need for additional steps such as edge detection when comparing images.

### Future considerations and advanced features

- Advanced imaging: The NeuRED Framework provides a versatile and user-friendly method of processing time-series radiography data with a focus on electrochemical devices, as demonstrated by technical examples above using various imaging setups and beamlines. Moreover, the *Modules* and *Utilities* packages can be used as a backbone of processing advanced imaging methods such as time-of-flight (ToF), grating interferometry, and pre-processing of 3D techniques. However, images obtained through these techniques are typically structured in a parallel (e.g., ToF imaging results in multiple images within a single pulse, each image having its own specific wavelength/energy range). Carreon Ruiz et al. demonstrated batch processing of such multiple data set based on the NeuRED Framework packages to study flow battery systems via polarized^38^ and Li-ion battery through ToF neutron imaging investigations^17^.
- Large volume handling: The use of the NeuRED Framework for advanced imaging modes, in which high frame rate is required, is limited by computing speed and management of a large volume of data. A Python environment allocates a single core for each Notebook by default. To make better use of multiple cores, parallel computing can be implemented within a Notebook using existing open packages, such as *joblib* or *multiprocessing*. An alternative, straightforward approach is to sub-group images and execute multiple Notebooks at the same time. In this case, each group must contain less than 15 GB of images to prevent a kernel crash and performance bottleneck. In contrast, for standard neutron imaging based on scintillator-CCD coupling, assuming each image is about 8 MB (2048 pixel × 2048 pixel, 16 bits), it is unlikely that computing speed becomes an issue, even for high flux imaging facilities in the ILL^39^ and ESS^40^.
- On-site processing: Another possible add-on feature is on-site processing capability. As demonstrated by examples above, it is difficult to discern changes in the samples without fully processing the images. On-site processing allows the users to optimize samples and conditions simultaneously for image acquisition during a beamtime. For standard time-series processes, this can be implemented simply by looping *batch_proc* periodically. Since the name and structure of images and folders are kept consistent between the source and destination directories, each time *batch_proc* undergoes an iteration it cross-checks for existing images in the destination and processes new images only. However, all necessary reference images (including DC, OB, BB, and dry) must be provided at the beginning of the processing of each sample, and the Notebook must be executed from top to bottom before initiating autonomous processing. The example script is provided at the end of Supplementary 3.
- Merging final images: The final image processed by using the NeuRED Framework is a single frame, cropped to the specified ROI, and saved in FITS format, with all processing steps are documented. To improve statistical reliability and overall quality, multiple images must be merged by using average or median projections. The *simple_merge* processor enables merging all images within a specified directory; therefore, it is not suitable for merging final images based on a defined time frame or every n-th frame, where n is specified by the user. Moreover, in time-series radiography, a sample is subjected to varying conditions (e.g., temperature, pressure, flow rates, and moisture). To facilitate the merging of final data, a time-based merging function, *ts_list_merge*, is introduced in the end of Supplementary 3. This function compares time information from metadata with a provided text file to generate a merging list. Example formatting for the text file is also included in Supplementary 3. If measurement conditions are recorded in the metadata, *ts_list_merge* can be adapted to match and merge data based on those conditions.
- Unit tests: In software development, unit tests are generally incorporated to verify critical functions and ensure stability and reproducibility. While the notebook-driven and interactive design of NeuRED presents challenges for conventional unit testing, particularly given the diverse nature of neutron imaging datasets, targeted unit tests for key functions (e.g., input/output magic selectors, processors, and scattered background computation) will be implemented in future releases. This will help ensure long-term robustness and support extensions to more advanced modalities, such as wavelength-resolved imaging.

## Conclusion

The NeuRED Framework offers a versatile, user-friendly, and comprehensive platform for processing time-series neutron radiographs, specifically designed for operando studies of electrochemical devices. By incorporating advanced image processing techniques, such as 3D white spot filtering, intensity correction, and registration, the Framework ensures high accuracy, reproducibility, and reliability of results. Its successful application to diverse systems, including fuel cells, half-cell Li-ion coin batteries, and high-pressure gas-liquid interactions, highlights its adaptability and robustness.

Open-source environments and modular architecture allow for high-level customization and scalability to a variety of imaging techniques, not just limited to neutrons. Furthermore, the *Modules* and *Utilities* can serve as a backbone for processing other types of experiments, in which the raw data consists of digitalized pixels, such as scattering techniques. The integration of metadata documentation ensures transparency and reproducibility of image processing, facilitating collaboration and promoting the adoption of neutron imaging techniques within the broader scientific community.

## Methods

### Structure of the neured framework

**Fig. 9:**
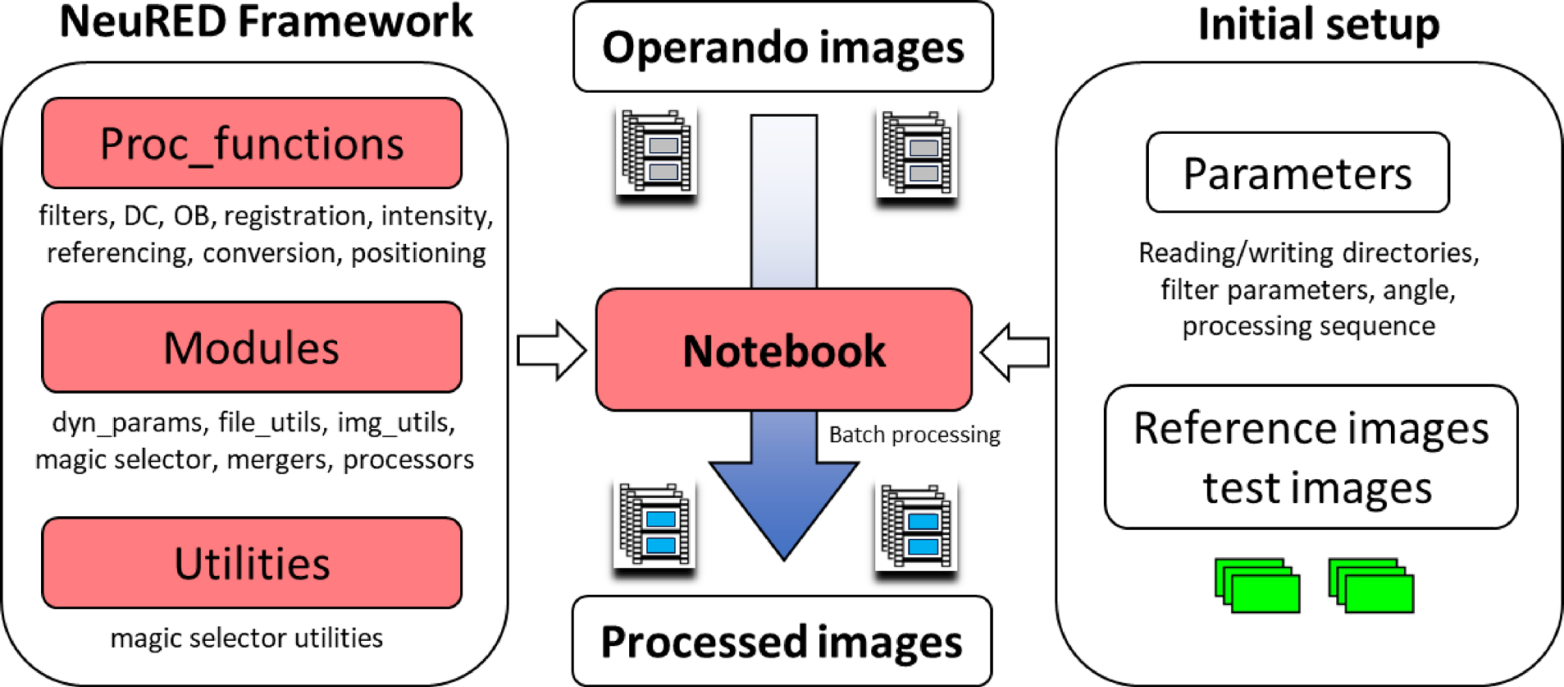
A schematic illustrating the structure of the NeuRED framework and its application to data processing. The red-filled boxes represent components provided within the framework. A master notebook is included, offering detailed explanations of key algorithms and instructions for execution. Before initiating batch processing, users must complete the initial setup, which includes determining processing parameters, preparing reference images, and verifying the quality of the test images. The DC and OB are the acronyms for dark current and open beam, respectively.

The NeuRED Framework was initially developed at the Paul Scherrer Institute (PSI) to process images from the Swiss Spallation Neutron Source (SINQ) : Imaging with COld Neutrons (ICON)^41^NEUtron Transmission RAdiography (NEUTRA)^42, ^and the Beamline for neutron Optics and other Approaches (BOA)^43^. The NeuRED Framework is designed for use in Jupyter Notebook, an open-source web-based interactive computing platform with Python as the core programming language. In comparison to other Python-based integrated development environments (IDEs), Jupyter Notebook provides a streamlined, web-style interface that allows users to sequentially navigate through data processing steps. This interface presents a significant advantage for users with limited programming experience. The core functionalities are organized as a collection of Python packages (.py) that are imported in the Notebook. If necessary, additional functions can be written and implemented directly within the Notebook, for example, to facilitate efficient debugging and rapid modifications. The overall structure of the Framework is presented in Fig. 9.

The core of the NeuRED Framework consists of three primary packages: *Proc_functions*, *Modules*, and *Utilities*. The *proc_functions* package contains a collection of image-processing routines, including filtering, dark current correction, and conversion to material thickness. The *Modules* package provides fundamental functionalities of the Framework, such as reading and writing images and files, as well as the magic selector and processors. Detailed explanations of the magic selector and processors are provided above (Results and Discussion: NeuRED Framework and its advantage). The Utilities package contains supporting functions for the magic selector.

Image processing is executed within a Jupyter Notebook, for which a demo Notebook file (.ipynb) is provided within the NeuRED Framework. In the demo, a detailed pipeline of the processing workflow is provided, including step-by-step explanations and an overview of the structure. In summary, users must first define initialization parameters (e.g., filtering parameters) and specify input/output directories. Subsequently, reference images, such as dark current (DC), open beam (OB), scattered background (SBKG), and reference/dry images, are processed fully before operando images. Then, a test processor is executed to ensure the quality of operando image processing, which is followed by batch processing of all images.

### Neutron imaging setup and samples

**Fig. 10:**
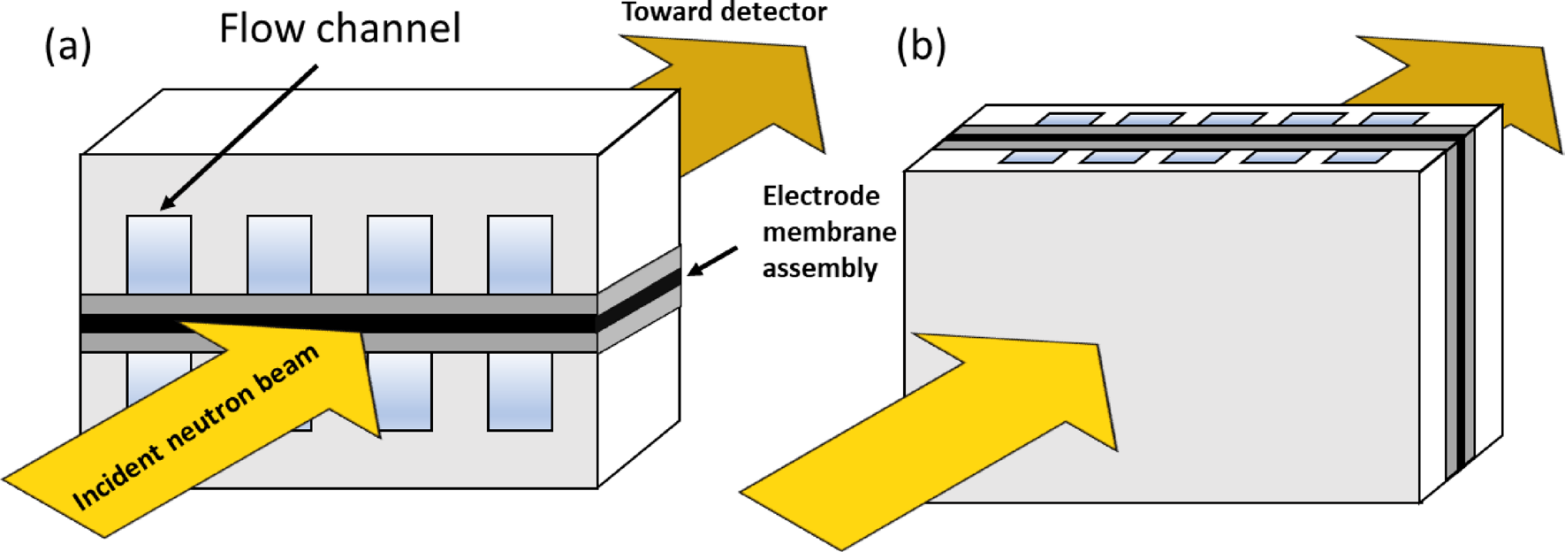
Schematics of the in-plane and through-plane imaging configurations for neutron radiography of fuel cells. (**a**) In-plane imaging: the MEA and the plane of the cell are aligned parallel to the incident neutron beam. (**b**) Through-plane imaging: the cell plane is oriented perpendicular to the neutron beam.

The neutron radiographs presented in this work were acquired at the NEUTRA at the Paul Scherrer Institute (PSI)^42^. A conventional scintillator-based digital camera, e.g., Andor iKon-L (Oxford Instruments), was used to achieve the effective spatial resolutions ranging from 10 to 50 μm, depending on the respective field of view (FoV) and imaging configurations used. For neutron imaging investigations of electrochemical devices, a custom single cell is typically employed. Given its sheet-layered structure, two imaging configurations are used: in-plane and through-plane, as illustrated in Fig. 10.

For in-plane imaging, the layered structure, such as the MEA in fuel cells, is aligned parallel to the incident neutron beam. This configuration allows for independent quantification of changes within individual layers before and after operation, which enables the study of product accumulation in porous layers. In contrast, through-plane imaging involves orienting the cell perpendicular to the beam. This approach offers higher spatial resolution and is compatible with larger cell areas (up to several hundred cm²); however, it does not provide detailed information about phenomena within individual components, as the beam traverses all layers of the cell. Therefore, through-plane imaging is typically employed to investigate significant changes in neutron transmission, such as water accumulation in the fuel cell flow field (on the scale of hundreds of micrometers to millimeters)^44^ or lithium (⁶Li) plating and stripping in Li-ion batteries^45^. In general, processing in-plane radiographs presents more challenges than through-plane imaging, particularly during the referencing step. Sample movements between reference and operando images, can lead to severe artifacts at material boundaries with contrasting attenuation and thickness.

**Table 1:** Overview of the samples and their respective imaging configurations used in the experimental setup. CCD: Andor Ikon-L. Scientific complementary metal-oxide-semiconductor (sCMOS): Orca flash 4.0 (Hamamatsu). In the resolution column, estimated effective Spatial resolutions are provided.

	Hardware	Imaging config.	Spatial resolution	Exposure time	Detector config.	Phenomena
Polymer electrolyte fuel cell	Custom ^46,47^	in-plane through-plane	50 μm 10 μm	5s 60s	Midibox/tilted^20^ with CCD	Water in flow channels and porous media
Li-ion battery	CR2032	in-plane	15 μm	30s	Taper^35^ with CCD	Lithiation and de-lithiation, bubble formation
High-pressure liquid-gas cell	Custom^36^	through-plane	50 μm	30s	Midibox with sCMOS	Gas absorption in the liquid, interfacial tensions

The samples and corresponding imaging setups serving as examples in this work are summarized in Table 1. Custom fuel cell and liquid-gas cell systems were designed to enhance neutron transmission by using aluminum and polytetrafluoroethylene (PTFE) and minimizing the thickness of components in the beam path. In the case of the battery study, a CR2032 coin cell was configured as a half-cell (with lithium metal and a copper electrode).

## Electronic supplementary material

Below is the link to the electronic supplementary material.

Supplementary Material 1

## Data Availability

The NeuRED framework is available in the Python Package Index (PyPI) repository, https://pypi.org/project/neured/. Please refer to Supplementary 1 for installation guide. The test data through-plane fuel cell images) is available in the Zenodo repository, Lee, J. (2025). Test data for NeuRED Framework (fuel cell) [Data set]. Zenodo.. The image datasets used in the Section: Demonstration of NeuRED Framework is available from the corresponding author on reasonable request.
