# Supplementary material for: Framework for processing operando neutron radiography of energy devices
Source: Sci Rep. 2025 Jul 16;15:25835. doi: 10.1038/s41598-025-09425-w (PMC12267630; doi:10.1038/s41598-025-09425-w)
Supplement: Supplementary file 1 — Supplementary Material 1 [file 41598_2025_9425_MOESM1_ESM.zip › Supplementary 1.pdf]

Supplementary 1

Framework for Processing Operando Neutron Radiography of Energy Devices

J. Lee[1,*], E. R. Carreon Ruiz[1], A. Kaestner[1], P. Trtik[1], M. Strobl[1], P. Boillat[1,2,*]

[1] PSI Center for Neutron and Muon Sciences, Paul Scherrer Institute, 5232 Villigen PSI, Switzerland
[2] PSI Center for Energy and Environmental Sciences, Paul Scherrer Institute, 5232 Villigen PSI, Switzerland

[*]Corresponding Author E-mail Address: jongmin.lee@psi.ch; pierre.boillat@psi.ch

1. Introduction

The Neutron Radiography of Electrochemical Devices (NeuRED) framework is a Python-based package for processing operando neutron radiograms, with a focus on normalization to quantify physical changes. Target applications include, but are not limited to, fuel cells, batteries, and electrolysers. Key advantages over existing tools include metadata tracking for processing steps and parameters, interactive user input, parameter optimization, and batch processing. These features enhance data integrity and reproducibility—critical requirements in electrochemical research.

The package is structured into three main components: *Proc_functions* (image processing), Modules (file and image utilities, processing tools), and Utilities (including the Magic Selector). While *NeuRED* is designed for use in Jupyter Notebook—allowing users to interactively process their own data following provided guidelines—it can also be run in any standard Python IDE without loss of core functionality. However, Jupyter-specific features (e.g., nbextensions for navigation and usability) will be disabled outside the notebook environment.

The demo Jupyter Notebooks provide step-by-step explanations for each processing function, offering a clear introduction to the framework. The accompanying publication in Scientific Reports (2025, XXXX) outlines the key features and practical use cases. For inquiries, please contact jongmin.lee@psi.ch or pierre.boillat@psi.ch.

2. Installation

> IMPORTANT: The NeuRED framework is designed for Python 3.12 (or later) and Jupyter Notebook 6.x (recommended <7.0). While Python 3.12 reflects the typical environment at the time of release, Jupyter 6.x is required due to compatibility issues with nbextensions used in the demo notebooks. Specifically, Jupyter 7.0 and above do not support certain features—such as collapsible headings and table of contents—that enhance user navigation. These limitations affect convenience only and do not impair the core functionality of the framework.

Before installing the NeuRED framework, ensure that Python (preferably version 3.12) and Jupyter Notebook (preferably version <7.0) are installed. If other versions are already in use, it is strongly recommended to create a dedicated environment for NeuRED. For example, using the Anaconda Prompt:

```
conda create -n neured_env python=3.12.3 #creates environment “neured_env” with Python 3.12.3
conda activate neured_env                #activates neured_env
pip install notebook==6.5.7              #install Jupyter Notebook 6.5.7
```

Further instructions for configuring Jupyter Notebook are provided in Section 2.4. The NeuRED framework itself can be installed directly from PyPI.

2.1. Installation using *pip* in Python environment

The framework can be installed using the command below. All required dependencies are specified and will be installed automatically:

pip install neured

If installed in the base environment, the package will be located in the following directory:

C:\Users\<<user_name>>\AppData\Roaming\Python\Python312\site-packages\neured

If installed in the neured_env environment, the package will be located in:

C:\Users\<<user_name>>\.conda\envs\neured_env\Lib\site-packages\neured

This installation method allows users to import the full NeuRED framework in Python using:

*from neured.framework.magic_selectors import **
*from neured import **

2.2. Configuring Jupyter Notebook

2.2.1 nbextension install

To enable Jupyter-specific features, install the nbextension package as follows. Ensure you are in the correct environment (e.g., neured_env):

pip install jupyter_contrib_nbextensions
jupyter contrib nbextension install –user

2.2.2 JavaScript files for Jupyter Notebook

In the directory "C:\Users\<*username*>\.jupyter", create a folder named "*custom*" (if it does not already exist), and copy the following files into it:

a. …neured\utilities\msel_utilities\custom.js
b. …neured\utilities\msel_utilities\msel_utilities.js

The default installation paths for these files are provided in Section 2.1.

Note: If a custom folder with an existing custom.js file is already present (which is uncommon), do not overwrite it. Instead, append the contents of *neured\utilities\msel_utilities\custom.js* to the existing *custom.js* file.

2.2.3 Configure Chrome as the default browser

This can be configured in Windows Settings under System > Default Apps (see Figure S1).

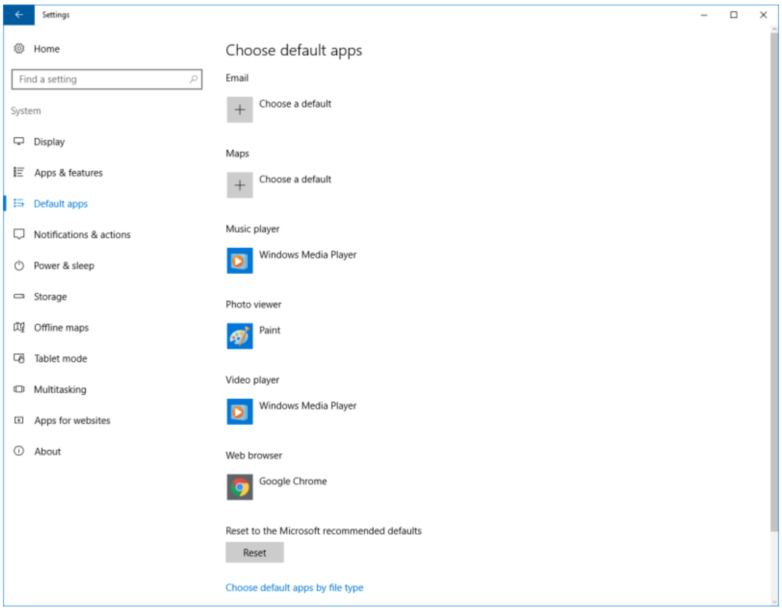

Figure S1. How to configure Chrome as the default browser.

Note: The processing notebook is compatible with other browsers; however, some shortcut buttons may not function as intended outside of Chrome.

2.2.4 Start Jupyter Notebook

If you are using the dedicated environment (e.g., *neured_env*), launch Jupyter Notebook by running the following command in the terminal: *jupyter notebook*

2.2.5 nbextension configuration

In the “nbextensions” tab within the Jupyter Notebook interface, enable the following options as seen in Figure S2:

a. Collapsible Headings
b. Table of Contents (2)
c. Export Embedded HTML

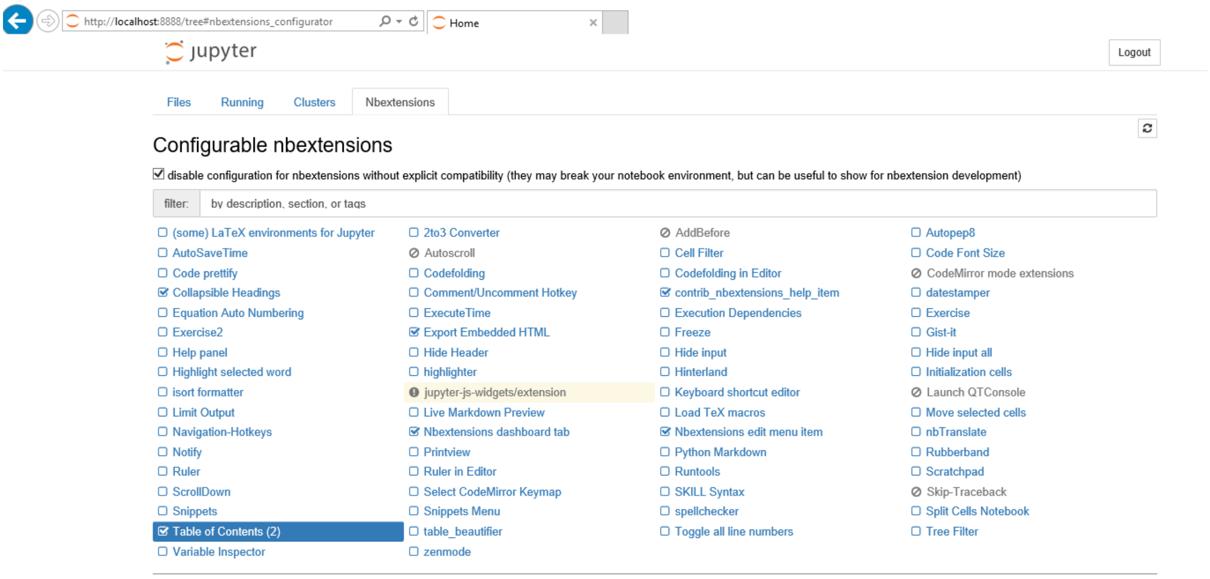

Figure S2. How to configure Chrome as the default browser.

On the “Table of Content (2)” options (Figure S3):

a. activate the option starting with “Skip h1 headings from numbering …”
b. Set the “Maximum level of nested sections …” parameter to 6

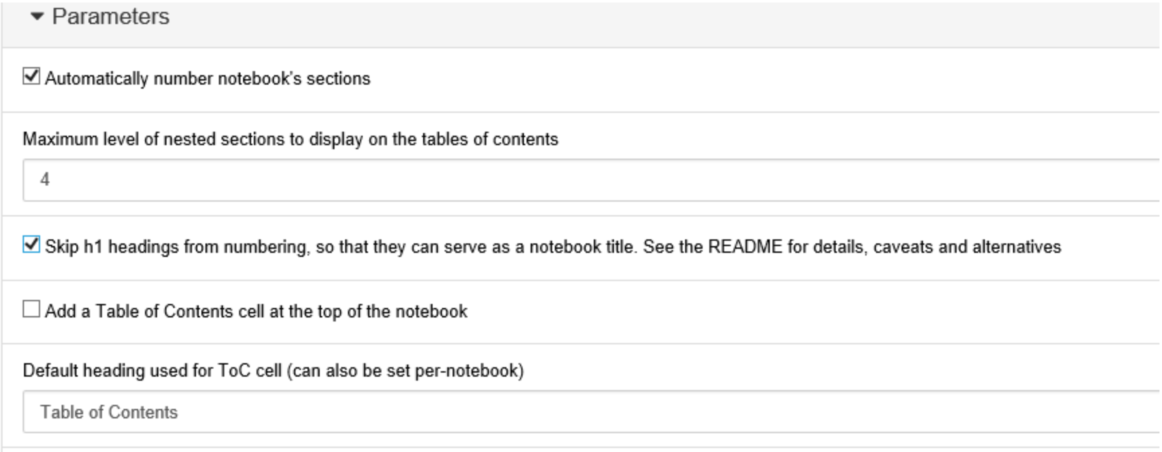

Figure S3. Setup for the Table of Content in nbextension panel.

2.2.6. Open demo notebook

Open a Jupyter notebook—for example, one located in the demo folder within the neured directory. If the installation was successful, you should see additional buttons labeled "Magic Selectors" in the left-side toolbar as illustrated in Figure S4.

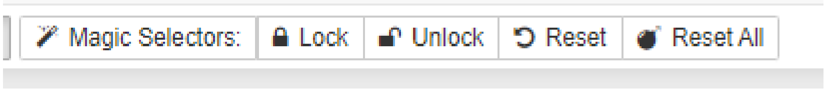

Figure S4. Additional buttons for magic selectors in the Jupyter Notebook.

Note: The demo notebooks are located in *...neured\demo_notebooks* as described in Section 2.1. You can navigate to this directory using the cd command in the terminal, or alternatively, copy the demo files to a preferred working directory.

2.3. Demonstration data

Demonstration data is available on Zenodo at DOI: 10.5281/zenodo.15019312. The dataset includes through-plane neutron radiograms of polymer electrolyte fuel cells, which are used in the accompanying demonstration notebooks.

3. Demo Jupyter Notebook (tutorial)

The package includes two demo notebooks: *NeuRED_Notebook_Demo_3Dfilter.ipynb* and *NeuRED_Notebook_Demo_withSBKG.ipynb*. The former provides a basic workflow featuring 3D filtering and intensity correction, while the latter demonstrates advanced features such as image registration and scattered background correction. If the installation is successful, the page below will be shown (Figure S5).

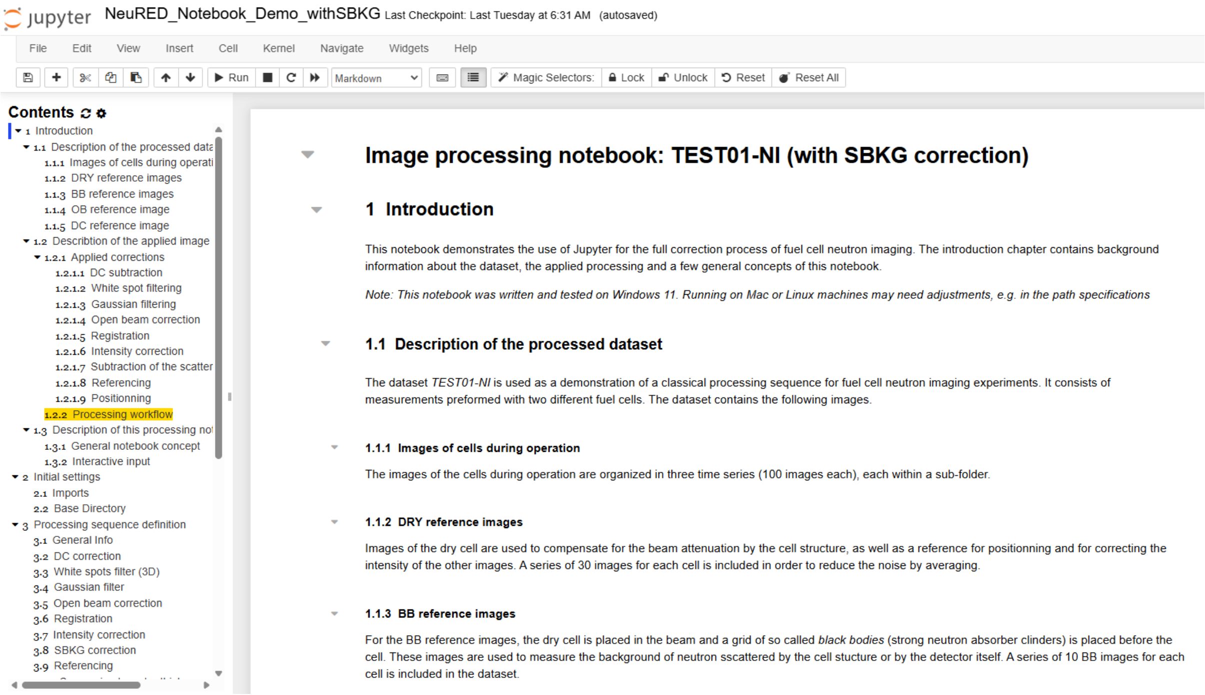

Figure S5. The first page of demonstration notebook (NeuRED_Noteboke_Demo_withSBKG.ipynb) opened on Jupyter Notebook.

Both notebooks are commented in detail to provide a guideline and serve as a tutorial. It is recommended that users go through the notebook cell-by-cell to understand process streamline and to get familiar with the NeuRED framework specific tools, such as magic selector. At the same time, image processing is directly performed on the notebook; the readers may modify the notebook as necessary.

4. A summary of the main modules and key functions

In Table S1, the backbone functions of the Neured framework are summarized. For the functions and processes directly related to image processing is defined in the demo notebook.

framework	dyn_params.py	● dynpar_lut() – Reads a dynamic parameter from a lookup table (e.g., for matching file tags to values).
	file_utils.py	● file_list(), change_ext(), add_tag(), get_tag(), change_tag(), file_index() – File name manipulation utilities.<br><br>● write_results(), read_file() – File I/O.
	img_utils.py	● get_img(), write_img(), crop_img(), get_3d_img(), write_3d_img() – Basic image handling.<br><br>● oversample_img() – Subpixel resampling of 2D images.<br><br>● img_render() – False-color rendering with optional masking.<br><br>● cmap_from_file() – Load color maps from CSV definitions.<br><br>● get_img_timestamp(), get_img_exposure() – Header metadata extractors.<br><br>● img_render_scale(), prep_img_for_display(), show_img(), img_roi_selector() – Display helpers.
framework	magic_selectors.py	● select_basedir(), select_fileopen(), select_roi(), select_multiple_rois() – Jupyter-integrated GUI selectors (e.g., base folder, ROIs).<br><br>● par_str_to_dict(), msel_load_image(), select_filesave_fits() – Misc utilities for interactive parameterization.
	mergers.py	● mrg_medproj(), mrg_avgproj(), mrg_avgproj_nan(), simple_merge(), multi_merge() – Projection and merging utilities.<br><br>● merge_headers(), load_tb_data(), ts_list_merge(), tag_based_mrg_list() – Multi-file alignment and merging logic.
	parameters.py	● set_params(), param(), get_all_params() – Set/get global or scoped parameters.<br><br>● set_group_scope(), set_general_scope() – Parameter grouping support.
	processors.py	● single_proc(), batch_proc(), batch_rendering(), batch_analyse() – Core processing logic.<br><br>● exec_start(), exec_end(), exec_proc(), test_proc(), consolidate() – Control flow and diagnostics.

		● load_img_params(), load_dyn_params(), load_imgdir_params(), cleanup_img_buffer() – Image + parameter loaders.<br><br>● get_proc_img(), get_neighbor_imgf(), get_neighbors_stack() – File-series navigation helpers.
Proc_functions	align.py	● img_align() – Image alignment (e.g., to correct drift or reposition frames).
	base_proc.py	● int_corr() – Intensity correction core (used by demo notebooks).
	filters.py	● ro_filter_2D(), ro_filter_3D() – Rolling average filters.<br>● ro_auto_threshold() – Adaptive thresholding for mask generation.
Proc_functions	positionning.py	● skew() – Applies affine skew transform to images.<br><br>● img_get_shifts() – Calculates pixelwise shifts to straighten elongated features.<br><br>● img_straighten() – Applies shift field to correct tilt/skew.
	resolution.py	● get_lsf() – Extracts the line spread function (LSF) from edges.<br><br>● fit_lsf() – Fits LSF using a Voigt function.<br><br>● voigt() – Analytical Voigt profile / MTF calculator.<br><br>● meas_resol() – (Internal helper for resolution evaluation).
	sbkg.py	● create_sbkg() – Constructs a scattered background image for correction.

Table S1. A summary of modules and main functions in the NeuRED framework.

5. Related publication

We kindly ask users to acknowledge the use of the NeuRED framework by citing the following publication:

Lee, J., et al. *Scientific Reports* (2025), XXXX.
