# Supplementary material for: Framework for processing operando neutron radiography of energy devices
Source: Sci Rep. 2025 Jul 16;15:25835. doi: 10.1038/s41598-025-09425-w (PMC12267630; doi:10.1038/s41598-025-09425-w)
Supplement: Supplementary file 1 — Supplementary Material 1 [file 41598_2025_9425_MOESM1_ESM.zip › Supplementary 2.pdf]

Supplementary 2

Framework for Processing Operando Neutron Radiography of Energy Devices

J. Lee[1,*], E. R. Carreon Ruiz[1], A. Kaestner[1], P. Trtik[1], M. Strobl[1], P. Boillat[1,2,*]

[1] PSI Center for Neutron and Muon Sciences, Paul Scherrer Institute, 5232 Villigen PSI, Switzerland
[2] PSI Center for Energy and Environmental Sciences, Paul Scherrer Institute, 5232 Villigen PSI, Switzerland

*Corresponding Author E-mail Address: jongmin.lee@psi.ch; pierre.boillat@psi.ch

	Input	Output	Purpose	Example code
**test_proc**	multiple images	display only	parameters testing, displaying results	test_proc(<br>src = param('test_img_dir'),<br>seq = seq_corr_ref,<br>proc_to='filtered',<br>roi = param('test_roi'),<br>test_param = 'ws_filter_size',<br>test_values = ([1,1,1], [3,3,3], [5,5,5]),<br>nimg = 1,<br>ncols = 3<br>)
**simple_merge**	multiple images	single image	merging a multiple images into a single image after processing	simple_merge(<br>src = param('ob_dir'),<br>dst = param('ob_imgf'),<br>merger = mrg_avgproj,<br>seq = seq_corr_ref,<br>proc_to = 'filtered'<br>)
**single_proc**	single image	single image	single image processing	single_proc(<br>src = param('bb_imgf'),<br>dst = param('sbkg_imgf'),<br>seq = [create_sbkg],<br>BB_mask_imgf = param('msk_imgf')<br>)
**batch_proc**	mutiple images	multiple images	batch image processing	batch_proc(<br>src = param('src_dir'),<br>dst = param('dst_dir'),<br>seq = seq_corr_ref<br>)

Table S2. Overview of processers of the NeuRED Framework.
